# A new method for measuring dopamine in the presence of uric acid employing a carbon paste electrode modified with the UiO-66 metal organic framework-graphene oxide nanocomposite

**DOI:** 10.5599/admet.2593

**Published:** 2025-02-04

**Authors:** Azhar Hameed Gatea, Aseer Shakir Ajel, Raed Muslim Mhaibes

**Affiliations:** 1 Department of Pathological Analytics Science, College of Applied Medical Science, Shatrah University, Thi-Qar 64001, Iraq; 2 Department of Biochemistry, College of Medicine, Misan University, Misan, Iraq

**Keywords:** Voltammetry, chemically modified electrodes, real sample analysis, electrochemical sensor

## Abstract

**Background and purpose:**

Dopamine has an impact on the cardiovascular, endocrine, renal, and central neurological systems. Electrochemical techniques are becoming more and more popular among researchers as a way to assess dopamine and uric acid levels.

**Experimental approach:**

Using electrochemical techniques, a new Universitet i Oslo MOF (UiO-66)-graphene oxide nanocomposite-modified carbon paste electrode was created to investigate the electrooxidation of uric acid and dopamine as well as their combinations. At the redesigned electrode, uric acid and dopamine were detected concurrently in a very sensitive way using differential pulse voltammetry (DPV).

**Key results:**

Dopamine DPV peak currents increase in a linear fashion at doses between 0.05 and 600.0 μM.

**Conclusion:**

Uric acid and dopamine levels in urine and dopamine injection samples may be determined with the help of the proposed sensor, which is reasonably priced and performs well.

## Introduction

Arvid Carlsson made the discovery of dopamine, one of the neurochemical transmitters in the brain [1]. The cardiovascular, endocrine, renal, and central neurological systems are all impacted by this drug [1]. Furthermore, it controls a number of physiological processes, including mental cognition, emotion, movement, behaveour, memory, learning, and attention [1]. Therefore, depression, schizophrenia, neurodegenerative disorders and addiction can all be brought on by an imbalance in dopamine levels in the human brain [1]. To track the amount of dopamine in the body, researchers must thus develop a simple, sensitive, and selective technique of dopamine detection. Research indicates that uric acid, eliminated through blood and urine, is one of the primary byproducts of purine metabolism in human bodies. Numerous illnesses, including hyperuricaemia, gout, and chronic renal disease, are brought on by extremely high levels of uric acid [2]. Due to their significant biological significance and crucial role in human metabolism, it has been shown that dopamine may be detected simultaneously with uric acid. Because they coexist in human fluids, the precision of the determination drastically drops in the combination sample; thus, it is crucial to keep an eye on their concentration. The relevant publications revealed that various analytical methods, such as chemiluminescence, spectrophotometry, colorimetry, high-performance liquid chromatography (HPLC), and electrophoresis, effectively detect uric acid and dopamine [3,4].

It is remarkable how widely accepted and approved the aforementioned approaches are, even though they are labour-intensive and need costly equipment and skilled technical professionals. Because electrochemical techniques are faster, more sensitive, and easier to use, researchers are becoming more interested in using them to assess the levels of dopamine and uric acid in biological fluids and pharmaceutical substances [5]. However, due to the lower potential fluctuations between oxidation peaks, it would be difficult to differentiate between dopamine and uric acid in electrochemical determination. Their distinctiveness would be limited by using common electrodes. Therefore, it would be essential to use a modified electrode to accurately measure the quantities of uric acid and dopamine [5].

The ability of chemically - modified electrodes (CMEs) to speed up the electrode process by drastically lowering the overpotential in comparison to an unmodified electrode is one of its most important features. By enabling a very selective coordination-based interaction between electron mediators and the target analytes, the electrodes can greatly increase selectivity of the electroanalytical procedures [5].

Electrochemical research has extensively used carbon paste electrodes (CPEs) [6]. Their success may be attributed to a number of features, such as their huge potential window, low ohmic resistance, and simplicity of modification [6]. However, increasing the electrodes' selectivity for identifying a particular analyte remains the primary obstacle. Because they may directly modify an electrode's chemical makeup, CMEs are an intriguing tool [6]. With the retention of the electroactivity of the analyte at the electrode surface, the electrode may be altered for enhancing the overall analytical conditions, signal overlapping shift and separation, initiation or amplification of the electrochemical signal, and/or minimization of the overpotential with the use of electrocatalysts [6]. Only a few chemical reactions should be expected, and the modifier should not be electroactive inside the window of interest. For every target species, the selectivity of the CME will be determined by one or more modifiers added to the structure's surface. While adsorption at the carbon particles may occasionally render this undesirable effect ineffective, modifiers should be somewhat insoluble in all solutions the sensor will come into contact with to avoid electrode "bleeding" [7].

The ease with which CPEs may be altered is among its best features. This is a result of CPE's highly adsorptive, well-developed surface. CPEs can be altered using a number of strategies. Several methods are available, including the most widely used one of mechanically adding dry modifiers to the paste [7], dissolving in the binding liquid, which entails soaking the carbon in the modifier and then drying it out before using it as an electrode, and in situ modification, which entails the modifier sticking to the surface of plain CPE and making it possible to identify the analyte in the solution [8].

The domains of electrochemistry and electroanalysis have made extensive use of room-temperature ionic liquids (RTILs) in recent years. Wider electrochemical windows, relatively strong ionic conductivity, low vapor pressure, and good chemical and thermal stability are some clear benefits RTILs have shown. Because of their broad electrochemical windows and high ionic conductivity, RTILs have been employed to create the modified electrodes [9].

Due to their higher conductivity than paraffin oil, ILs can be utilized to create paste electrodes for voltammetric studies. This is due to their ability to decrease the charging current while increasing the sensitivity and detection limit. Moreover, since they offer more active sites at the electrodes surface, nanoparticles with large specific surface area might offer a viable platform for sensor formation. We modified the carbon paste electrode using these two significant chemical types in consideration of the previously mentioned parameters [10].

Most electrodes, left unmodified, show considerable overpotential and low sensitivities, resulting in a build-up of fouling on the surface with time. The ability to electrochemically detect different analytes requires the use of modified electrode surfaces. The modification of an electrode can increase the degree of electron exchange between the electroactive species and electrode surfaces [11]. Consequently, considerable efforts have been made to fabricate better electrodes by using various materials and nanostructures. Some of the benefits of nanomaterials include higher surface areas, strong catalytic activity, more surface-active sites, and improved conductivity. These properties have the potential to enhance sensor stability and sensitivity significantly. Furthermore, they may act as catalysts to enhance the assessment of electrochemical reactions and improve the efficacy of electron transport [12].

As nanomaterials developed, functional material was incorporated into electrodes to increase their sensitivities. Metal-organic frameworks (MOFs) have received considerable attention in a wide range of applications due to their high specific surface areas and porous structure [13]. Several factors make UiO-66 noteworthy. These MOFs are in high demand due to their remarkable attributes, such as chemical and thermal stability, resistance to mechanical strain, and ease of regeneration.

Sturdy O-Zr bonds, a high coordination number, and strong linkages between non-mineral blocks and organic linkers define the UiO-66 structure. They are appealing choices for a variety of applications, including sensors, energy devices, gas adsorption, heterogeneous catalysis, and medicinal applications, due to their adaptability in both structure and function. According to recent research, combining MOFs with other nanostructures can greatly increase the durability and electrocatalytic potential of modified electrodes in addition to improving their electrical conductivity [13].

Graphene oxide (GO) and its derivatives, fullerene, mesoporous carbon, carbon nanotubes and other carbon nanostructures, are materials with a wide range of uses and very variable structure and characteristics [14,15]. Significant progress has been made in carbon nanostructures since graphene, one of the carbon allotropes, was discovered. Carbon atoms arranged in a 2-dimensional honeycomb pattern make up single-layer graphene sheets. Despite its many characteristics, GO is a feasible and useful material for a range of electrochemistry applications, such as energy storage and conversion, electrochemical sensing, *etc.*, due to its high electrical conductivities and surface areas [16-18].

The goal of the current project was to create and describe the UiO-66-GO nanocomposite. The UiO-66-GO nanocomposite-based sensor has a broad linear range, a low limit of detection, and excellent catalysis towards dopamine. The sensor's catalytic performance for determining dopamine in the presence of uric acid was also examined. Real urine and dopamine injection specimens were used to test the suitability of modified electrodes for sensor applications.

## Experimental

### Apparatus and chemicals

For the electrochemical experiments, the Autolab potentiostat/galvanostat was utilized. A platinum wire, an Ag/AgCl/KCl (3.0 M) and UiO-66-GO/IL/CPE served as the working, auxiliary, and reference electrodes, respectively. Using a Metrohm 710 pH meter, the pH was determined. We used double-distilled water to create each solution from scratch. All additional reagents, including analytical-grade dopamine and uric acid, were supplied by Merck. Buffer solutions were made with orthophosphoric acid and their salts.

### Synthesis of UiO-66-graphene oxide nanocomposite

A simple one-pot solvothermal method was used to create the UiO-66/GO nanocomposite. ZrCl_4_ (0.037 g) and terephthalic acid (0.026 g) were first combined and dissolved in 20 milliliters of N,N-dimethylformamide (DMF). To the previously stated solution, 2.5 ml of acetic acid was added dropwise while stirring to fully dissolve the precursors. In the next step, 0.00675 g of GO was ultrasonically dispersed in the previously described solution for an hour. After that, the resulting suspension was put in an autoclave and heated to 120 °C for eight hours. Following the solvothermal reaction, the sediments were collected, washed several times using ethanol and distilled water, and then vacuum-dried at 60 °C for 15 hours.

### Preparation of the UiO-66-GO/IL/CPEs

To manufacture the UiO-66-GO/IL/CPEs, 0.10 g of UiO-66-GO nanocomposite, 0.90 g graphite powder and approximately 0.70 ml of paraffin oil and 0.30 ml ionic liquid (n-haxyl-3-metylimidazolium hexafluorophosphate) were combined to manufacture the paste. The components were placed in a mortar and pestle before being amalgamated to procure a smooth texture, after which paste was introduced into the hollow of a glass tube approximately 10 cm long. The hollow section was connected to the copper wire to manufacture the electrical contacts. The surface of the carbon paste was polished using fine paper prior to each experiment. For the unmodified CPE, the procedure remained identical except that UiO-66-GO nanocomposite and ionic liquid were excluded.

### Procedure of real sample preparation

To make a dopamine solution, we start by taking 1 mg ml^-1^ dopamine ampoule and diluting it by 1 ml using 0.1 M PBS. Subsequently, the mixed grape solution is diluted to a total amount of 50 ml. After proper dilution, the solution is poured into a PBS and added to 25 ml flasks. A urine sample is then collected and put inside a refrigerator, where a 20 ml sample is put inside a centrifuge for 20 minutes at 1500 rpm. The new solution is put inside a volumetric flask and is diluted with PBS. Following the previous steps, uric acid alongside dopamine is added to the new sample. Once the final sample is created, based on the previously used methods and techniques, dopamine and uric acid concentrations are determined.

## Results and discussion

### Characterization of UiO-66/GO nanocomposite

**Figure 1.**
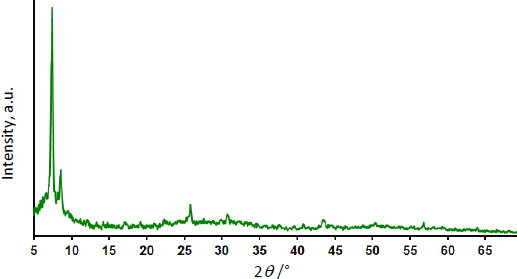
XRD pattern of UiO-66/GO nanocomposite

X-ray diffraction was used to determine the crystalline structure of the UiO-66/GO nanocomposite as it was formed (Figure 1).

The primary diffraction peaks in the nanocomposite's XRD pattern resemble those shown in prior studies for UiO-66 MOF [19-21]. It is just UiO-66 MOF that exhibits these diffraction peaks, the XRD pattern showed no peaks associated with GO.

**Figure 2.**
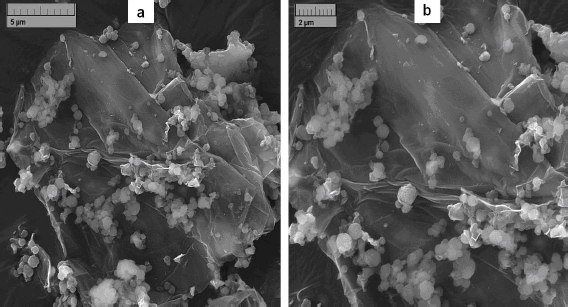
Images of FE-SEM for UiO-66/GO nanocomposite at different magnifications

FE-SEM was used to examine the surface morphologies of the generated nanocomposite. The FE-SEM pictures of the UiO-66/GO nanocomposite are shown in Figure 2. On the GO nanosheets, UiO-66 MOF formation was evident.

### Cyclic voltammetric study of dopamine oxidation

**Figure 3.**
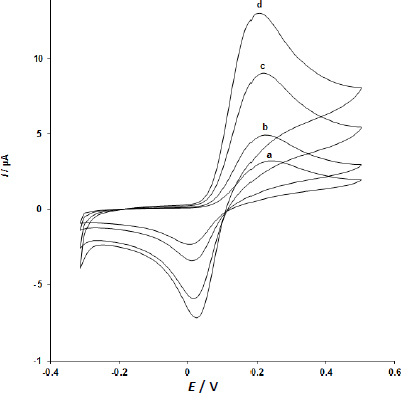
At the surface of the unmodified CPE(a), UiO-66/GO/CPE (b), IL/CPE (c), and UiO-66/GO/IL/CPE (d), there were CVs of 500.0 μM dopamine

Electrooxidation of 500.0 μM dopamine on bare CPE (curve, a), UiO-66/GO/CPE (curve, b), IL/CPE (curve c), and UiO-66/GO/IL/CPE (curve d) is shown by the cyclic voltammetric responses in Figure 3. The bare CPE peak potential is at 250 mV (curve a), while the anodic peak potential of the dopamine oxidation at UiO-66/GO/IL/CPE is evidently at 210 mV (curve d).

The anodic peak current at UiO-66/GO/IL/CPE is significantly higher than the value measured on bare CPE (curve a) when we look at dopamine oxidation at the two sites (curve d and a). The results clearly show a combination of graphite powders, UiO-66/GO nanocomposite, and IL improves the characteristics of dopamine oxidation.

**Figure 4.**
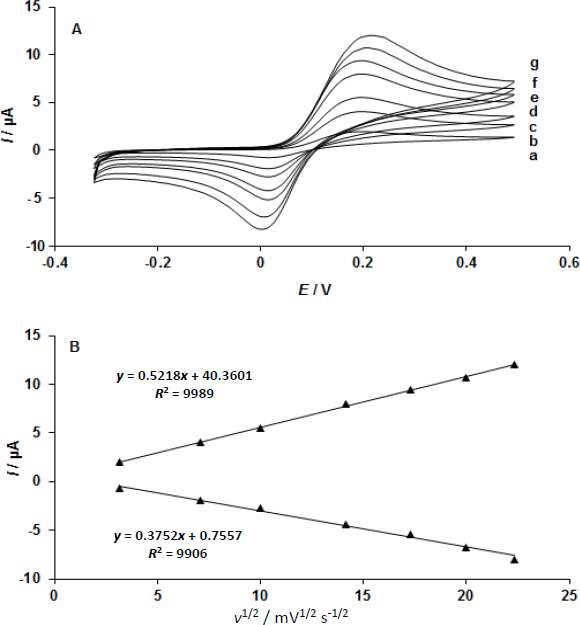
(A) UiO-66/GO/IL/CPE CVs at different sweep rates in 150.0 μM dopamine; curves a to g represent 10.0, 50.0, 100.0, 200.0, 400.0, and 500.0 mV s^-1^, respectively; (B) Cathodic and anodic peak current variation *vs. v*^1/2^

CV was used to examine the impact of potential sweep rates on dopamine electrocatalytic oxidations at the UiO-66/GO/IL/CPE (Figure 4A). In the 10.0–500.0 mV s^-1^ range, a linear plot of peaks height *I vs. v*^1/2^ was seen, indicating the process is diffusion-based as opposed to surface-controlled (Figure 4B).

### Chronoamperometric measurements

**Figure 5.**
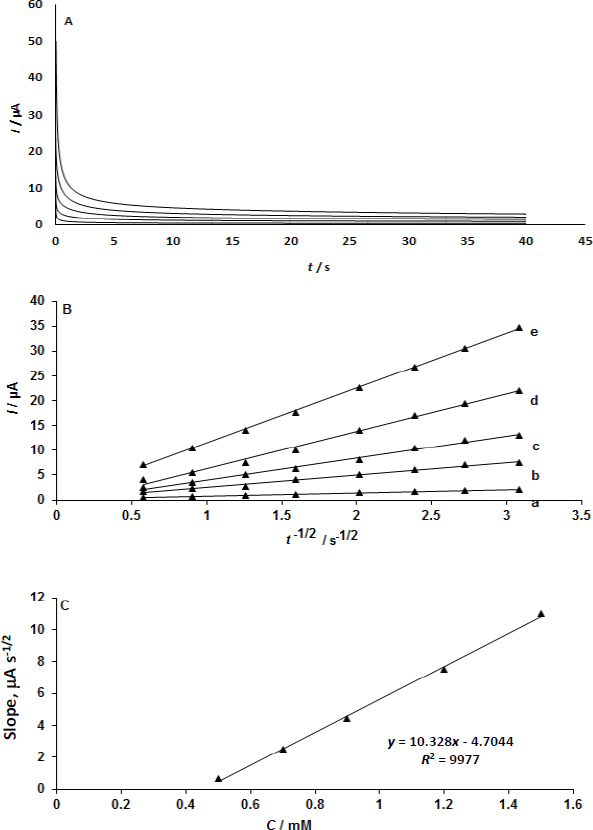
(A) chronoamperograms for various dopamine concentrations (0.5, 0.7, 1.0, 0.9, 1.2, and 1.5 mM of dopamine) were obtained at UiO-66/GO/IL/CPE. (B) chronoamperogram plots of *I vs. t*^-1/2^ were obtained; and (C) straight lines slopes was plotted against dopamine concentration.



Dopamine at UiO-66/GO/IL/CPE was determined using the given amperometric measurements in buffered aqueous solutions with various dopamine concentrations (Figure 5A). Cottrell equation (1) [22] is the equation that represents the current as the function of time, *t*, at the electrode under mass transport conditions.

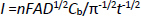

(1)


*I vs. t*^-1/2^ experimental plots were used, and Figure 5B shows the best fit for various dopamine doses. Plotting the slopes of the obtained straight lines against the concentration of dopamine was the next step (Figure 5C). The value of *D* was determined to be 110 cm^2^ s^-1^ using the Cottrell equation and the resulting slope. This value is in good agreement with previously obtained values of 1.52×10^−6^ [23] and 2.26×10^−6^ cm^2^ s^-1^ [24].

### Calibration plot and limit of detection

**Figure 6.**
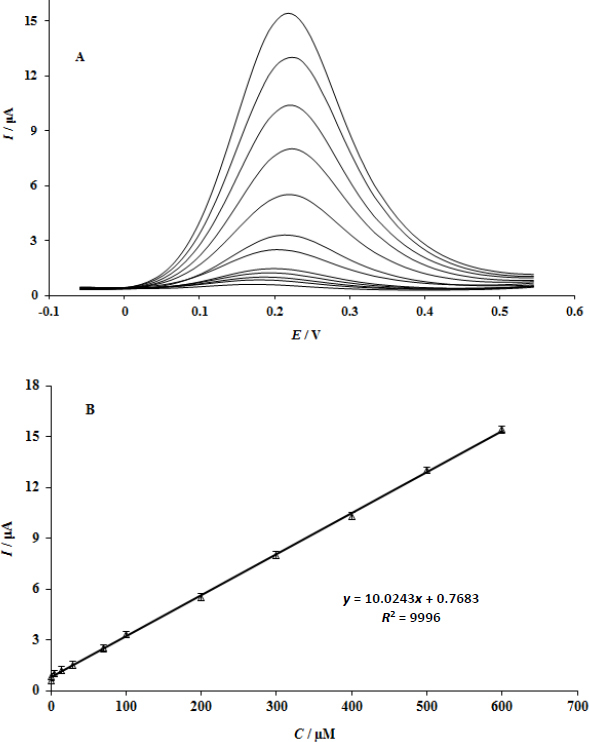
(A) shows the DPVs of UiO-66/GO/IL/CPE with varying dopamine concentrations. (B) A plot of the peak current in the 0.05 to 600.0 μM dopamine.

It is possible to determine the quantity of dopamine in solution by measuring the peak current of dopamine oxidation at the UiO-66/GO/IL/CPE surface. DPV tests were conducted using UiO-66/GO/IL/CPE with different doses of dopamine (Figure 6A). Peak current vs concentration of dopamine was shown as a linear segment in the concentration range of 0.05 to 600.0 μM (slope = 0.0243 μA. μM^-1^). 0.02 μM was found to be the detection limit (3*s*) (Figure 6B). In terms of dopamine measurement, this figure is better than a number of recent studies (1.22 [2] and 1.0 μM [23]).

### Simultaneous determination of uric acid and dopamine

**Figure 7.**
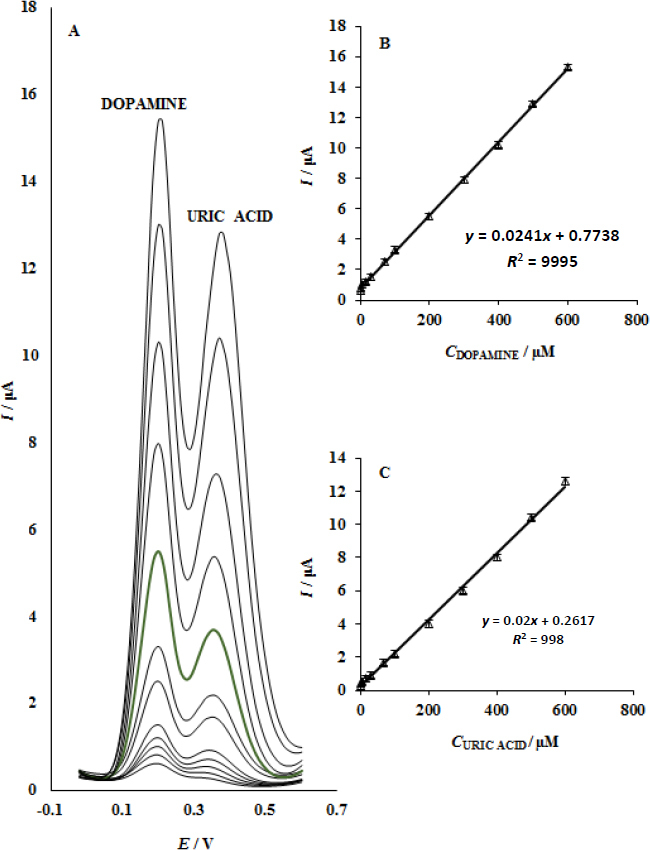
(A) DPVs of UiO-66/GO/IL/CPE with varying dopamine and uric acid concentrations in μM, arranged from inner to outer: 100.0+100.0, 200.0+200.0, 300.0+300.0, 400.0+400.0, 500.0+500.0, 0.05+0.05, 0.5+0.5, 5.0+5.0, 15.0+15.0, 30.0+30.0, 70.0+70.0, and 100.0+100.0, in that order. Plots of *I vs.* dopamine concentration (B) and *I* versus uric acid concentration (C)

The paper describes the application of the UiO-66/GO/IL/CPE to simultaneously measure uric acid and dopamine for the first time. There are no such cases where this tool has ever been used for such a purpose in any work. The uric acid electrochemical detection on bare electrodes is disturbed by dopamine presence. Uric acid and dopamine were varied simultaneously, and the DPVs were recorded to identify the two substances (Figure 7A). Figure 7A shows the voltammetric curve that displayed two peaks at 380 and 210 mV, which are attributed to uric acid and dopamine oxidation, respectively. This indicates that the application of the UiO-66/GO/IL/CPE enables the detection of both of them concurrently. The slope for dopamine oxidation was 0.0241 μA/μM. The reason is that this figure is so close to the value that the substance gives us, separately, without its presence or non-presence. At the UiO-66/GO/IL/CPE, it is possible to measure their concentration simultaneously without significant interferences.

### Uric acid and dopamine measurements in urine and dopamine injection samples

The samples of dopamine and uric acid in urine and dopamine from injection ones were also tested. The process recommended by the analytical applicability of the gadget was used in analyzing this procedure. Table 1 represents the outcomes of the two species' recognition in real samples. The trial showed that uric acid and dopamine recovered rather well. The procedure's repeatability was shown using RSD.

**Table 1.** UiO-66/GO/IL/CPE estimation of dopamine and uric acid in real samples (*n* = 5)

Sample	*C* / μM				Recovery, %		RSD, %	
	Spiked		Found					
	Dopamine	Uric acid	Dopamine	Uric acid	Dopamine	Uric acid	Dopamine	Uric acid
Dopamine injection	0	0	3.1	-	-	-	3.4	-
	2.5	5.5	5.7	5.4	101.8	98.2	2.7	3.0
	5.0	7.5	7.9	7.7	97.5	102.7	1.9	2.2
	7.5	9.5	10.4	9.6	98.1	101.0	2.5	2.6
	10.0	11.5	13.4	11.1	102.3	96.5	2.8	1.8
Urine	0	0	-	2.9	-	-	-	2.9
	5.0	3.0	5.1	5.8	102.0	98.3	3.6	2.2
	7.0	5.0	6.8	8.1	97.1	102.5	2.7	3.3
	9.0	7.0	9.4	9.8	104.4	99.0	1.7	2.0
	11.0	9.0	10.9	12.0	99.1	100.8	2.3	2.7

## Conclusions

This work examined the electrochemical characteristics of dopamine using CPE modified with IL and a UiO-66/GO nanocomposite. When peak current was plotted versus dopamine concentration, a linear segment with a LOD = 0.020 μM was found in the concentrations range of 0.05 to 600.0 μM. The DPV measurement of uric acid and dopamine in their combination solutions shows great selectivity because the updated electrode effectively resolves the overlapping voltammetric peaks of these two substances. Lastly, the altered electrode was investigated as a novel electrochemical sensor that can precisely and selectively identify dopamine and uric acid in actual samples.
